# Supplementary material for: Coherent hexagonal platinum skin on nickel nanocrystals for enhanced hydrogen evolution activity
Source: Nat Commun. 2023 Apr 27;14:2424. doi: 10.1038/s41467-023-38018-2 (PMC10140298; doi:10.1038/s41467-023-38018-2)
Supplement: Supplementary file 1 — Supplementary Information [file 41467_2023_38018_MOESM1_ESM.pdf]

Supplementary Information

Coherent Hexagonal Platinum Skin on Nickel Nanocrystals for Enhanced Hydrogen Evolution Activity

Kai Liu,[1#] Hao Yang,[2#] Yilan Jiang,[3,4#] Zhaojun Liu,[1] Shumeng Zhang,[1] Zhixue Zhang,[1] Zhun Qiao,[1] Yiming Lu,[2] Tao Cheng,[2*] Osamu Terasaki,[3,4] Qing Zhang,[3,4*] and Chuanbo Gao[1*]

[1]*State Key Laboratory of Multiphase Flow in Power Engineering, Frontier Institute of Science and Technology, Xi'an Jiaotong University, Xi'an, Shaanxi 710054, China.*

[2]*Institute of Functional Nano & Soft Materials (FUNSOM), Jiangsu Key Laboratory for Carbon-Based Functional Materials & Devices, Joint International Research Laboratory of Carbon-Based Functional Materials and Devices, Soochow University, Suzhou, Jiangsu 215123, China.*

[3]*Center for High-resolution Electron Microscopy (CħEM), School of Physical Science and Technology, ShanghaiTech University, Shanghai 201210, China.*

[4]*Shanghai Key Laboratory of High-resolution Electron Microscopy, ShanghaiTech University; Shanghai, 201210, China.*

[#]*These authors contributed equally to this work.*

**Email: gaochuanbo@mail.xjtu.edu.cn (C.G.), tcheng@suda.edu.cn (T.C.), zhangqing1@shanghaitech.edu.cn (Q.Z.).*

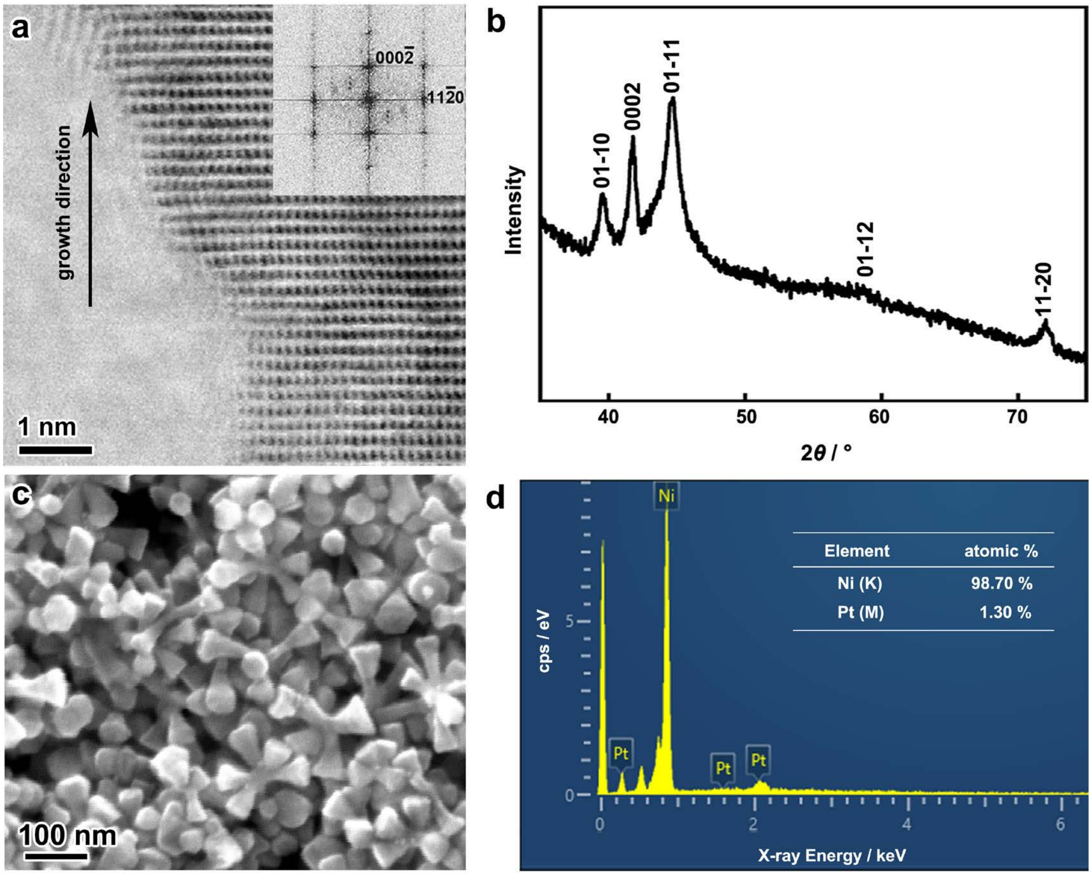

Supplementary Fig. 1 | Characterization of the *hcp*-Ni nanobranches. (a) HRTEM image. Inset: Fourier diffractogram. Zone axis, [1-100]. The nanobranch grows along the <0001> direction. (b) XRD pattern. (c) SEM image. (d) EDS analysis. Signals of Pt (1.3 atom%) were from the Pt seeds in the Ni nanobranches.

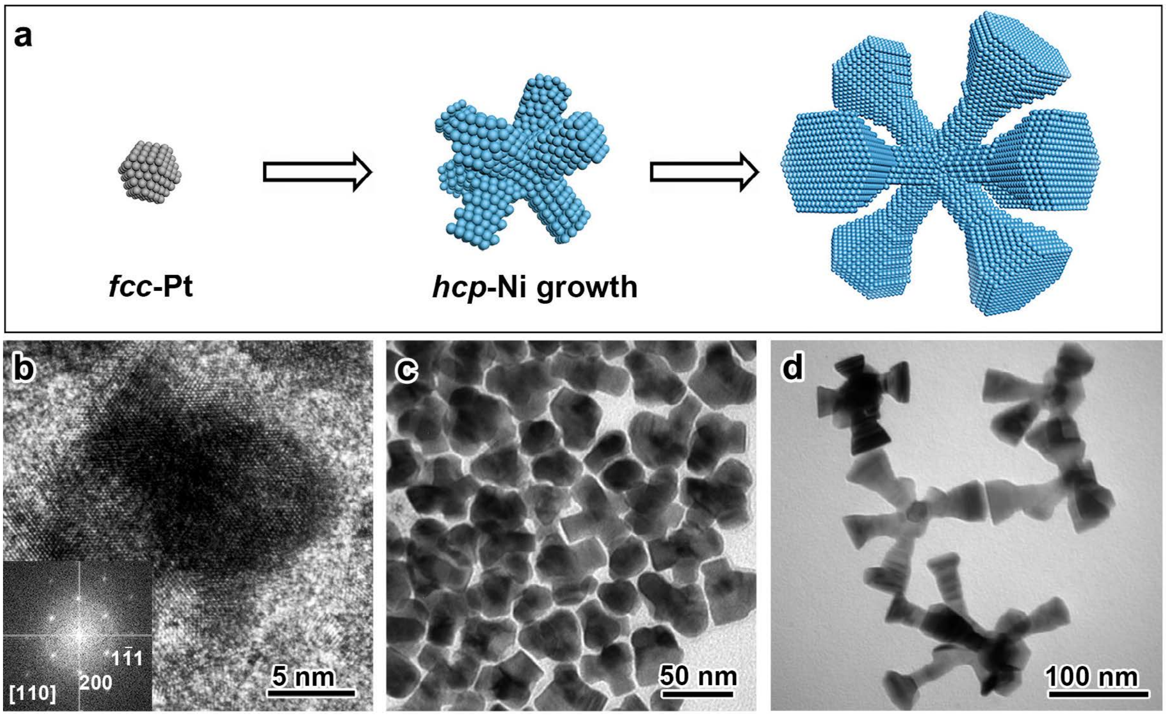

Supplementary Fig. 2 | Formation mechanism of the *hcp*-Ni nanobranches. (a) Schematic illustration of the synthesis. *fcc*-Pt nanocrystals were first formed, followed by the growth of *hcp*-Ni nanobranches in the <0001> direction on {111} facets of the Pt seeds. Due to the defects of the Pt seeds, the number of the {111} facets on the Pt seeds may vary, leading to different numbers of the branches from 5 to 9 in an individual *hcp*-Ni nanobranch. (b–d) TEM images of the intermediates obtained at different stages of the synthesis. Reaction time: (b) 210 min, (c) 230 min, and (d) 300 min. The intermediate obtained at 210 min are *fcc*-Pt nanocrystals, according to the lattice size and the Fourier diffractogram. The large size of the Pt particle (~10 nm) may result from the aggregation of the small seeds during high-speed centrifugation for collecting the intermediate for characterization.

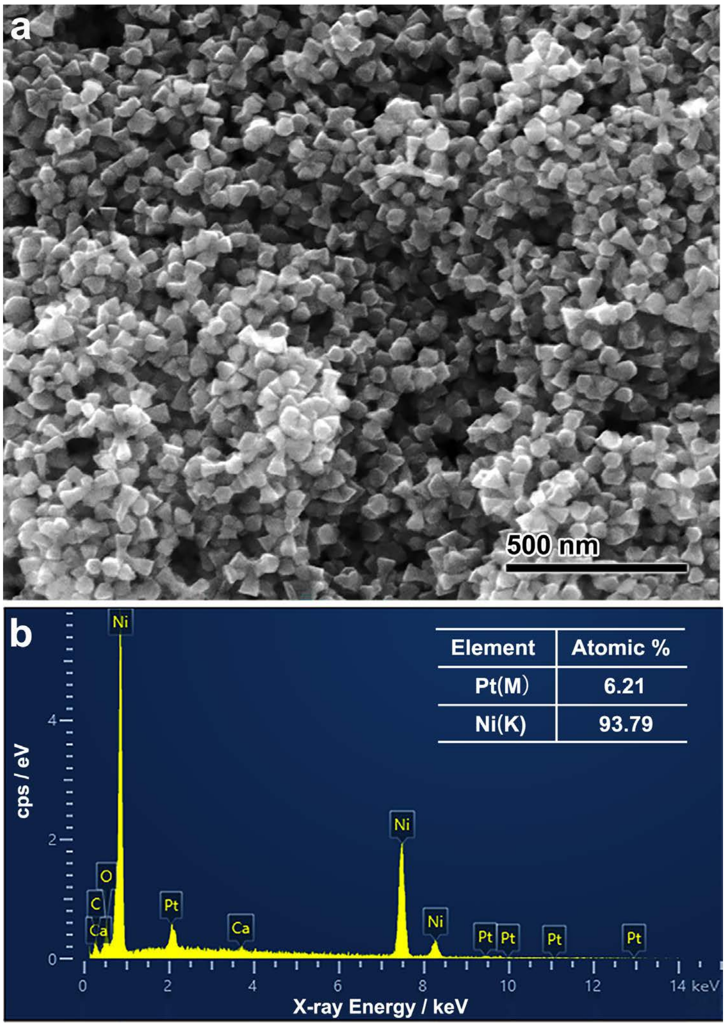

Supplementary Fig. 3 | Characterizations of the *hcp*-Ni@Pt-skin nanobranches. (a) SEM image. (b) EDS analysis.

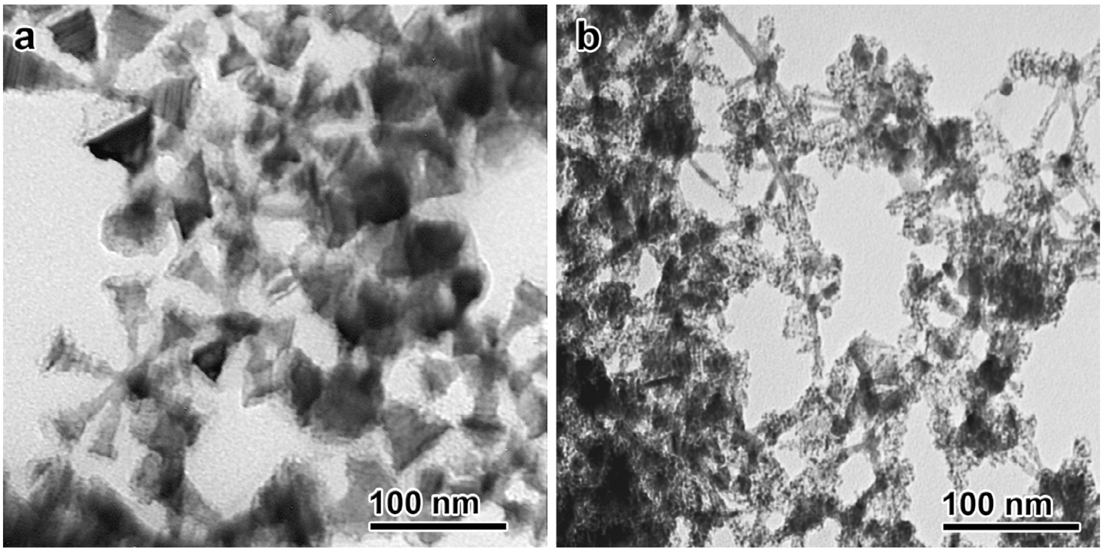

Supplementary Fig. 4 | Role of oleylamine in suppressing the galvanic replacement reaction in the synthesis. (a, b) TEM images of the products after the growth of Pt on Ni nanobranches without oleylamine in the precursor in a typical synthesis. The amounts of 0.1 M H_2PtCl_6 were 0.1 mL (a) and 0.3 mL (b), respectively. An etching of the Ni templates was observable, highlighting the critical role of oleylamine in suppressing the galvanic replacement reaction between the Ni templates and the Pt salt.

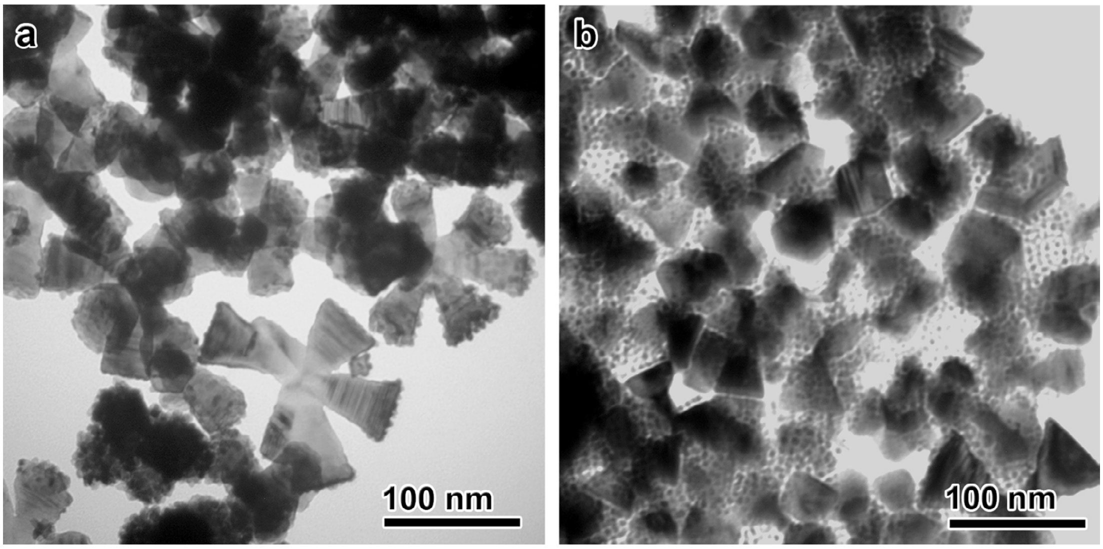

Supplementary Fig. 5 | TEM images of the products obtained from a typical synthesis with high injection rates of the H_2PtCl_6+oleylamine precursor. Injection rates: (a) 1.2 mL min^{-1}; (b) 2 mL min^{-1}. Pt islands were formed on the Ni nanobranches. Free nucleation of Pt was also observed. These results suggest that a slow injection rate of the Pt precursor is favorable for a layer-by-layer growth of Pt on the Ni nanocrystals.

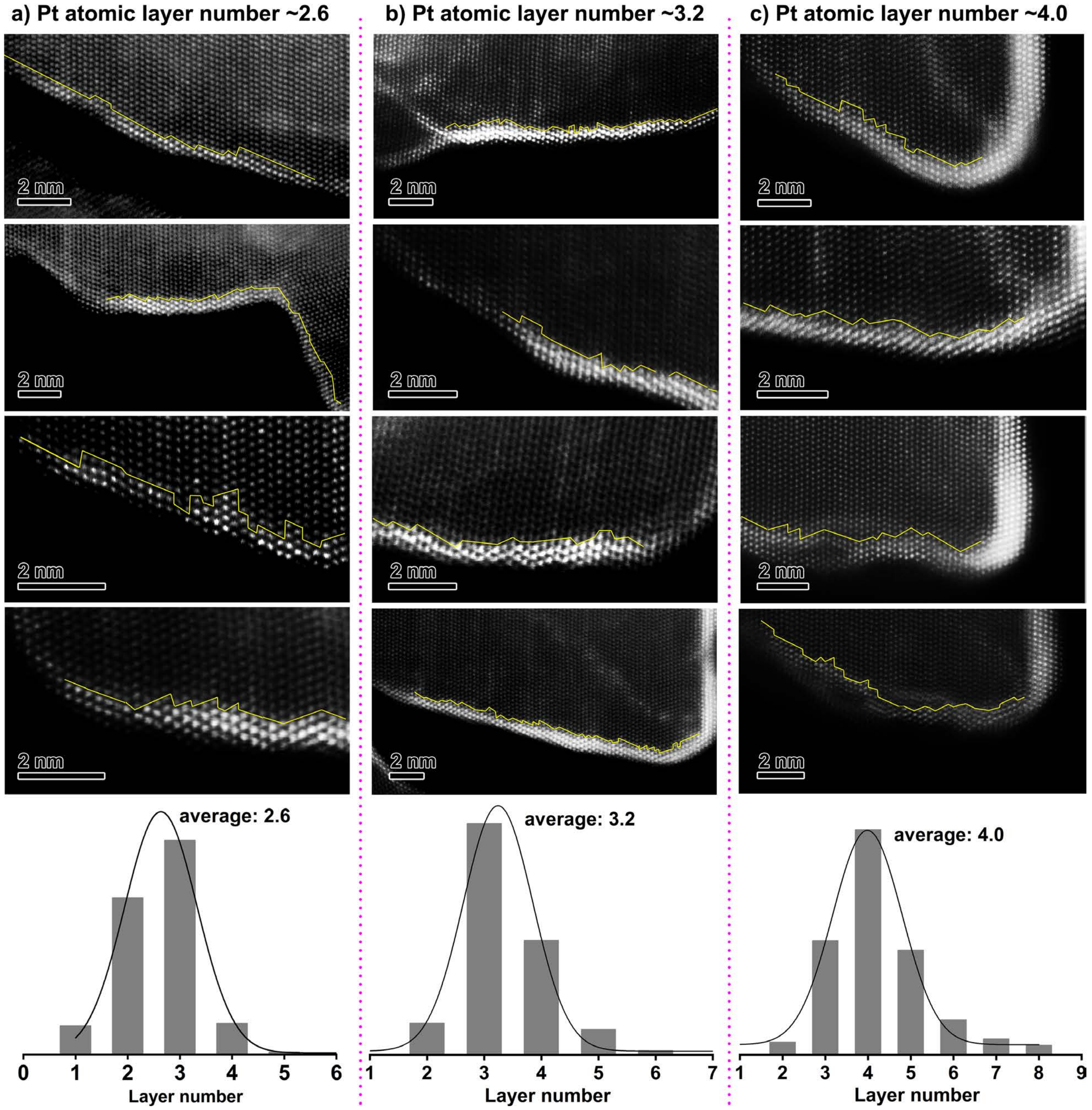

Supplementary Fig. 6 | C_s-corrected HAADF-STEM images of the *hcp*-Ni@Pt core-shell nanobranches with different atom layers of the Pt skins. Below the images: histograms of the atomic layer numbers of the Pt skins. (a–c) The average layer numbers of the Pt skins are 2.6, 3.2, and 4.0, respectively. Yellow lines indicate the core-shell boundary in each image.

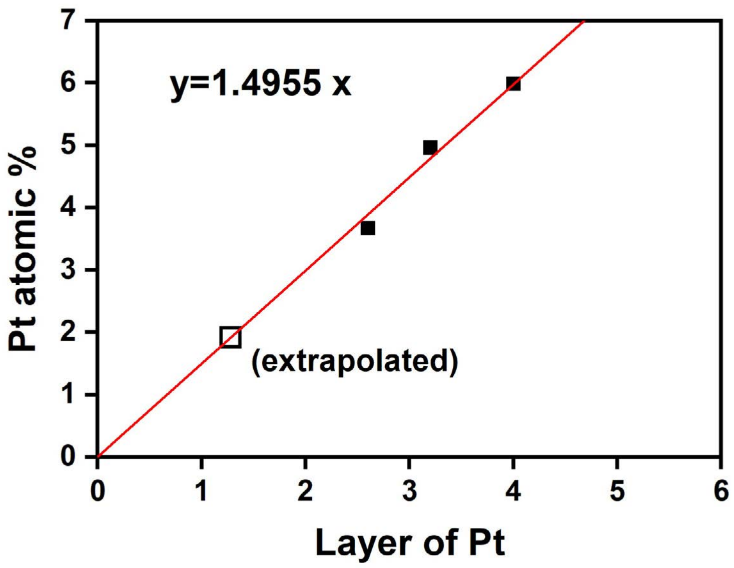

Supplementary Fig. 7 | Correlation between atomic fractions of Pt in the *hcp*-Ni@Pt core-shell nanobranches measured by ICP-MS and thickness of the Pt skins measured by HR-STEM. Note that the atomic layer number of 1.3 was estimated by extrapolation of the linear relationship, according to the ICP-MS results.

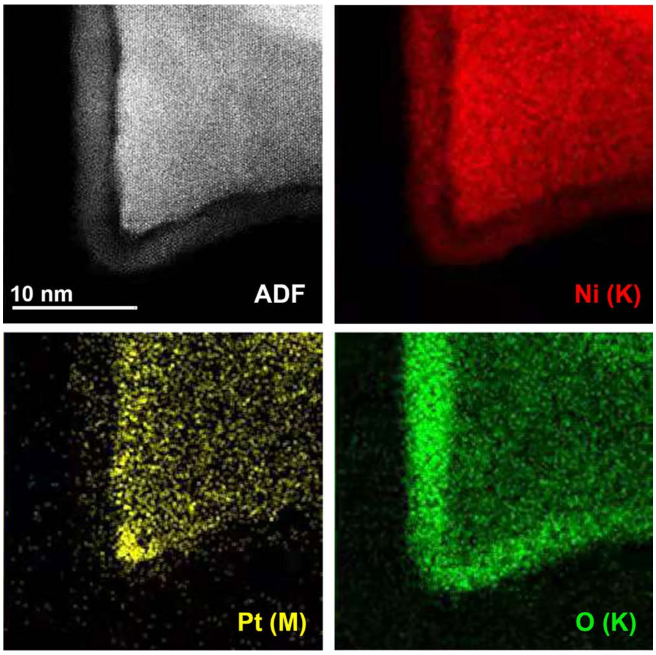

Supplementary Fig. 8 | EDS elemental mapping of the *hcp*-Ni@Pt$_{1.3L}$ nanobranches. The *hcp*-Ni@Pt$_{1.3L}$ nanobranches are prone to oxidation in ambient air. Therefore, the nanobranches are covered by a thick layer of NiO, which may block the active sites on the surface, leading to substantially decreased catalytic activity in the HER.

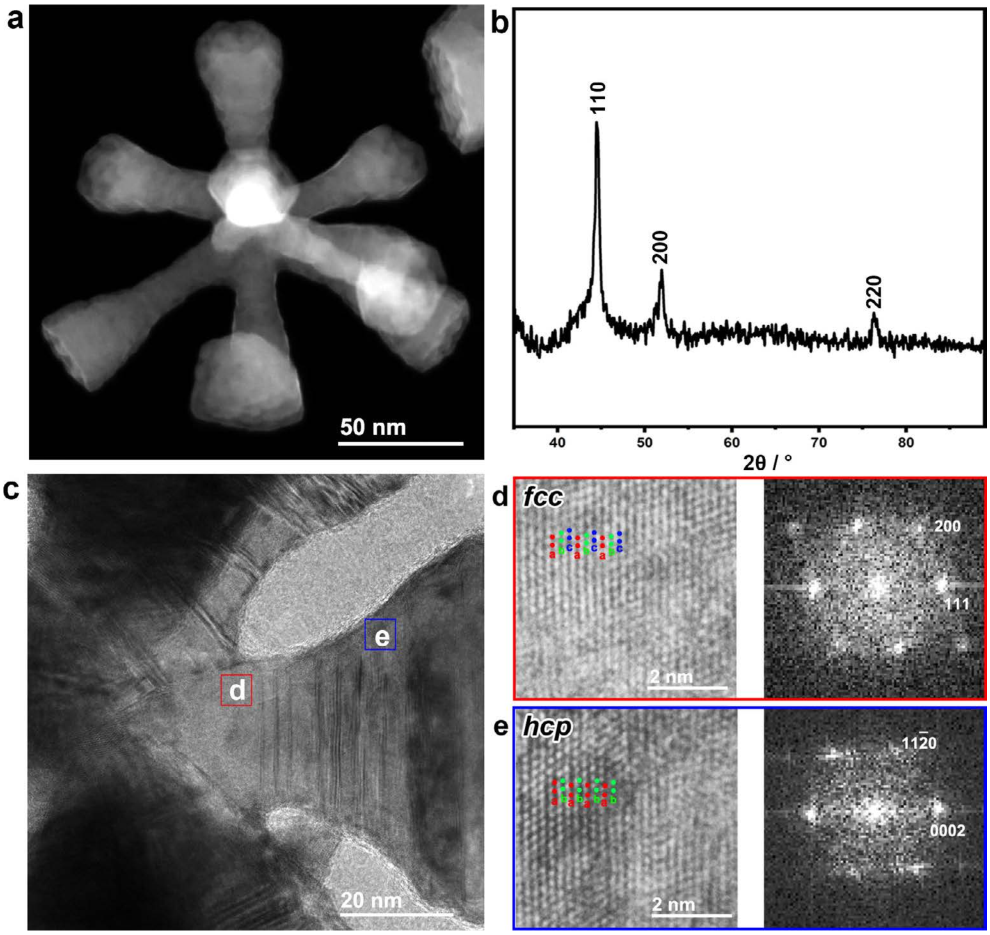

Supplementary Fig. 9 | Characterizations of the *fcc*-Ni@Pt$_{2.6L}$ core-shell nanobranches. (a) STEM image. (b) XRD pattern. (c) Low-magnification HRTEM image. (d, e) HRTEM images and Fourier diffractograms corresponding to the zones labeled in (c). The XRD pattern confirms that the *hcp*-Ni@Pt core-shell nanobranches are successfully transformed into the *fcc* phase by the thermal treatment. STEM image shows no obvious morphological change during the phase transformation. A high-contrast skin can be observed at the edge of the nanobranches, corresponding to Pt atoms, which confirms that the core-shell nanostructure has been retained during the thermal treatment. From the HRTEM images, we can clearly observe the *fcc* zones, although there are minor *hcp* zones remaining in the nanobranches. The XRD pattern as a whole-sample-based analysis confirms that the *fcc* phase is the major phase in the sample.

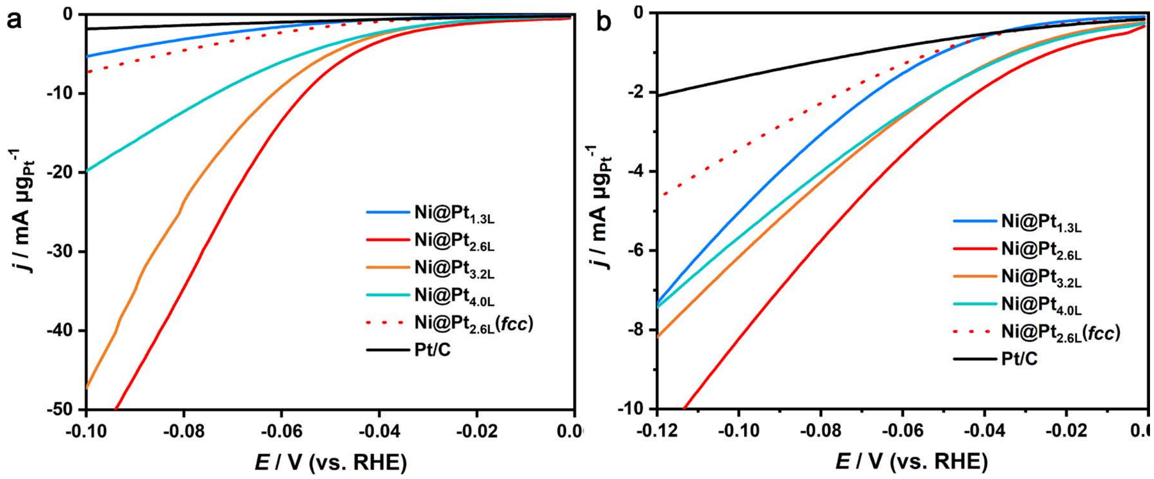

Supplementary Fig. 10 | LSV curves of different catalysts in the electrocatalytic HER at different *iR* compensation levels. (a) 95%-*iR* compensation. (b) Non-*iR* compensation. The solution resistances (*R*) with the catalysts of *hcp*-Ni@Pt$_{nL}$ (*n* = 1.3, 2.6, 3.2, 4.0), *fcc*-Ni@Pt$_{2.6L}$, and the commercial Pt/C were measured automatically by the CHI760e workstation to be 6.237, 4.114, 5.293, 5.181, 3.604, and 3.399 Ω, respectively. Pt loading, 1.5 μg; electrolyte, 1 M KOH; scan rate, 10 mV s^{-1}. The mass activity of *hcp*-Ni@Pt2.6L was measured to be 23.0 mA μg_{Pt}^{-1} at –70 mV with 95% *iR* compensation.

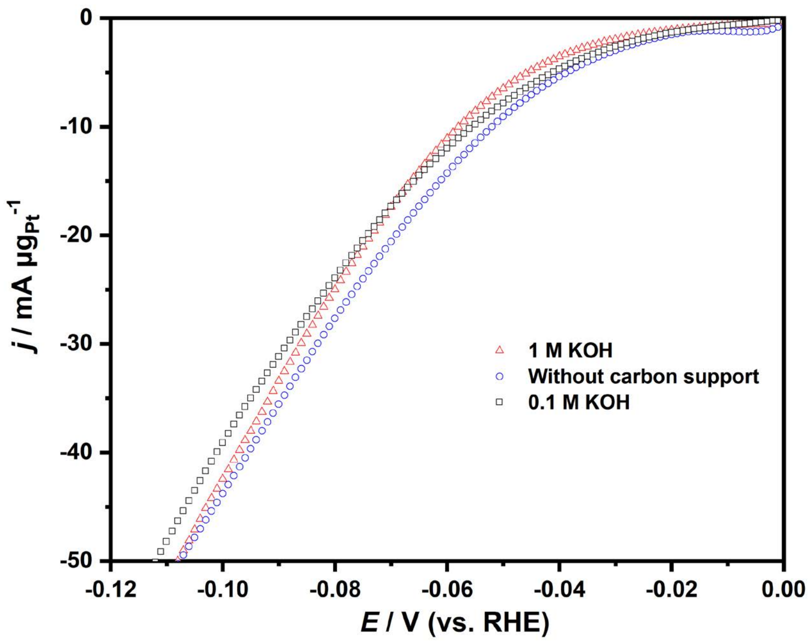

Supplementary Fig. 11 | LSV curves of the *hcp*-Ni@$Pt_{2.6L}$ catalyst in the alkaline HER under different conditions: measurements in 1 M and 0.1 M KOH with catalysts supported on carbon nanotubes, and measurement in 1 M KOH with catalyst without carbon support. Level of *iR* compensation: 90%. *R*: 4.313 Ω (measurement in 1 M KOH), 38.1 Ω (measurement in 0.1 M KOH), and 2.688 Ω (measurement in 1 M KOH, catalyst without carbon support). The mass activities at –70 mV were measured to be 17.4, 17.3, and 20.6 mA μg_{Pt}^{-1}, respectively. The HER activity of the nanobranches in 0.1 M KOH is included in Supplementary Table 1 for comparison with values reported in the literature. The result also suggests that the support (carbon nanotubes) does not impose a significant influence on the catalytic activity of the *hcp*-Ni@$Pt_{2.6L}$ nanobranches.

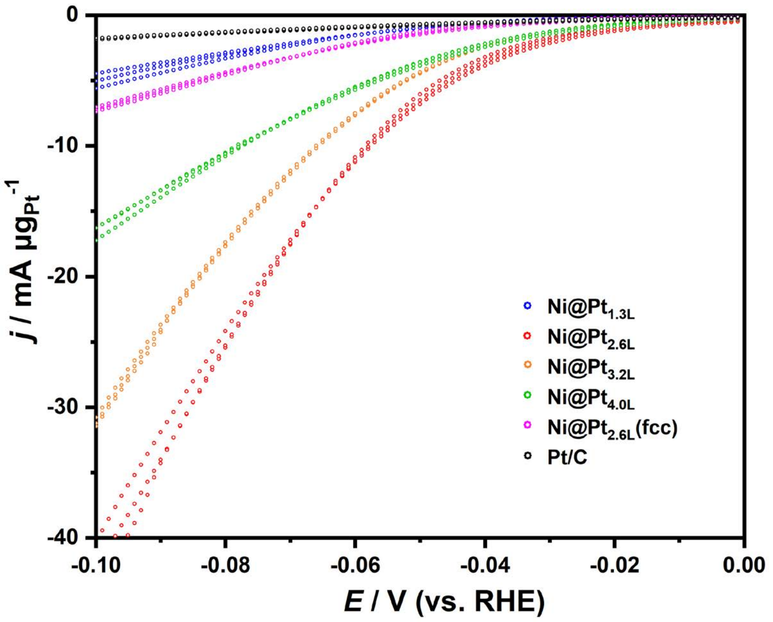

Supplementary Fig. 12 | LSV curves of the *hcp*-Ni@Pt$_{n\mathrm{L}}$ catalysts from 3 parallel electrocatalytic HER measurements. The *iR* compensation levels, 90%. *R* values: Ni@Pt$_{1.3\mathrm{L}}$: 6.237, 6.415, and 5.454 Ω; Ni@Pt$_{2.6\mathrm{L}}$: 4.114, 5.217, and 4.455 Ω; Ni@Pt$_{3.2\mathrm{L}}$: 5.293, 5.351, and 5.431 Ω; Ni@Pt$_{4.0\mathrm{L}}$: 5.181, 5.787, and 4.937 Ω; Ni@Pt$_{2.6\mathrm{L}}$ (*fcc*): 3.604, 4.649, and 3.998 Ω; Pt/C: 3.399, 3.399, and 3.032 Ω.

Supplementary Table 1 | HER activities of the *hcp*-Ni@Pt nanobranches and typical Pt nanocrystal-based catalysts reported in the literature.**

Catalysts	Electrolyte (KOH)	Loading ($\mu g_{Pt}\ cm^{-2}$)	Mass activity ($mA\ \mu g_{Pt}^{-1}$)	Tafel slope ($mV\ dec^{-1}$)	Exchange current density ($mA\ cm^{-2}$)	Year	Ref.
hcp-Ni@Pt-skin	1 M	7.65	17.4@–70 mV	56	18.2 (normalized to electrode area)	This work	
	0.1 M	7.65	17.3@–70 mV	68	19.9 (normalized to electrode area)		
hcp-RuIrPt alloy nanoparticles	1 M	50*	0.877@–30 mV	22.3	-	2022	1
Ultrafine PtNiP nanowires	1 M	30.6	6.27@–70 mV	30	-	2022	2
Monoatomic Pt embedded *hcp*-Ni nanosheets	1 M	2.0	30.2@–100 mV	47	0.41 (normalized to ECSA)	2021	3
Single-atom Pt catalyst on $MoSe_2$	1 M	3.47	34.4@–100 mV	41	-	2021	4
Pt–Ni nano-thorn arrays	1 M	16	4.27@–50 mV	38	-	2021	5
$PtNi_5$ alloy nanoparticles	1 M	10	6.0@–70 mV	19.2	-	2021	6
Ni-Pt island nanoparticles	0.1 M	1.02	7.8@–250 mV	43	-	2021	7
Pt nanoparticles/Fe-doped $Ni(OH)_2$ nanosheets	1 M	7.36	15.2@–70 mV	25.6	-	2020	8

Single-atom-nickel modified Pt nanowires	1 M	2.0	11.8@–70 mV	60.3	-	2019	9
Pt islands on branched Ni nanoparticles	0.1 M	0.408	7.7@–70 mV	69	2.18 (normalized to ECSA)	2019	10
Octahedral PtNi-O nanostructure	1 M	5.1	7.23@–70 mV	78.8	-	2018	11
Lotus thalamus-shaped Pt-Ni nanocrystals	1 M	17	2.80@–70 mV	27	1.35 (normalized to electrode area)	2018	12
PtNi-S nanowires	1 M	15.3	4.9@–70 mV	114.7	-	2018	13
hcp Pt-Ni excavated nano multi-pods	0.1 M	7.65	3.03@–70 mV	78	1.65 (normalized to ECSA)	2017	14
Pt nanowires on nickel hydroxide	0.1 M	16.1	1.59@–70 mV	-	-	2015	15

* Including the mass of Ru, Ir, and Pt.

** It is difficult to directly compare the catalytic activities of materials reported in the literature, because the values depend on materials, operational methods and conditions, metal loadings, supports for catalysts, and so on. This table lists the values and key conditions for the measurements for generally surveying the activities of the previously reported catalysts. It is not intended for direct comparison purpose.

Evaluation of specific activities of the *hcp*-Ni@Pt$_{n\mathrm{L}}$ nanobranches in alkaline HER.

The specific activity (SA) is defined as the current density normalized to the electrochemically active surface area (ECSA) of the catalyst. The ECSA of Pt-based catalysts can be usually obtained by underpotential deposition of hydrogen in the potential range of 0.05–0.4 V vs. RHE. However, the Ni cores in the *hcp*-Ni@Pt$_{n\mathrm{L}}$ nanobranches are prone to oxidation at positive potentials, albeit with the protection of surface Pt. Therefore, it is difficult to obtain the ECSA values, and thus the specific activity, of the *hcp*-Ni@Pt$_{n\mathrm{L}}$ nanobranches. To overcome the difficulty in directly measuring the ECSA experimentally, we here adopt two alternative ways to estimate the ECSAs, and thus the specific catalytic activities, as follows.

(1) We assume Pt is grown on the Ni substrate as a uniform thin layer (atomic layer number, *n*). For this specific structure, the fraction of Pt atoms on the surface is 1/*n*. Given a constant mass of Pt, the number of Pt atoms on the surface (thus the specific surface area) is proportional to 1/*n*:

$$\mathrm{ECSA} \propto n^{-1}$$

$$\text{or } \mathrm{ECSA} = k\, n^{-1} \qquad \text{(eq. 1)}$$

Because the specific activity (SA) is related to the mass activity (MA) by the following equation:

$$\mathrm{MA} = \mathrm{SA} \cdot \mathrm{ECSA}$$

the specific activity can be derived as:

$$\mathrm{SA} = \mathrm{MA} \cdot \mathrm{ECSA}^{-1} = k^{-1} \cdot \mathrm{MA} \cdot n \propto \mathrm{MA} \cdot n \qquad \text{(eq. 2)}$$

Therefore, the value obtained by multiplying the mass activity (MA) of the *hcp*-Ni@Pt$_{n\mathrm{L}}$ nanobranches by the atomic layer number of the Pt skin (*n*) can be used as a measure of their specific activity (SA), with MA and *n* easily obtainable by electrochemical experiments and C_s-corrected HAADF-STEM imaging, respectively. The unit of the specific activity is mA·layer·μg^{-1}, which is a

variant of the traditional unit of mA cm^{-2}. Because the evaluation of the specific activity by this method involves few assumptions, we adopted this approach in Fig. 3d to reliably reflect the intrinsic HER activities of the *hcp*-Ni@Pt-skin core-shell nanobranches to be correlated to the atomic layer number of Pt in the nanobranches.

(2) Because the main exposing facets of the *hcp*-Ni@Pt$_{nL}$ nanobranches are {01-11} (Fig. 1), we can further estimate the ECSA of the nanobranches by involving a new parameter, i.e., the average area (A_0) each Pt atom occupies on the {01-11} facet. The *fcc* phase of the Pt layer at the top of the nanobranch was ignored in this calculation.

First, the A_0 value is calculated according to the following model and lattice parameters (a = b = 2.89 Å, c = 4.32Å; c/a = 1.49) experimentally determined by C_s-corrected HAADF-STEM.

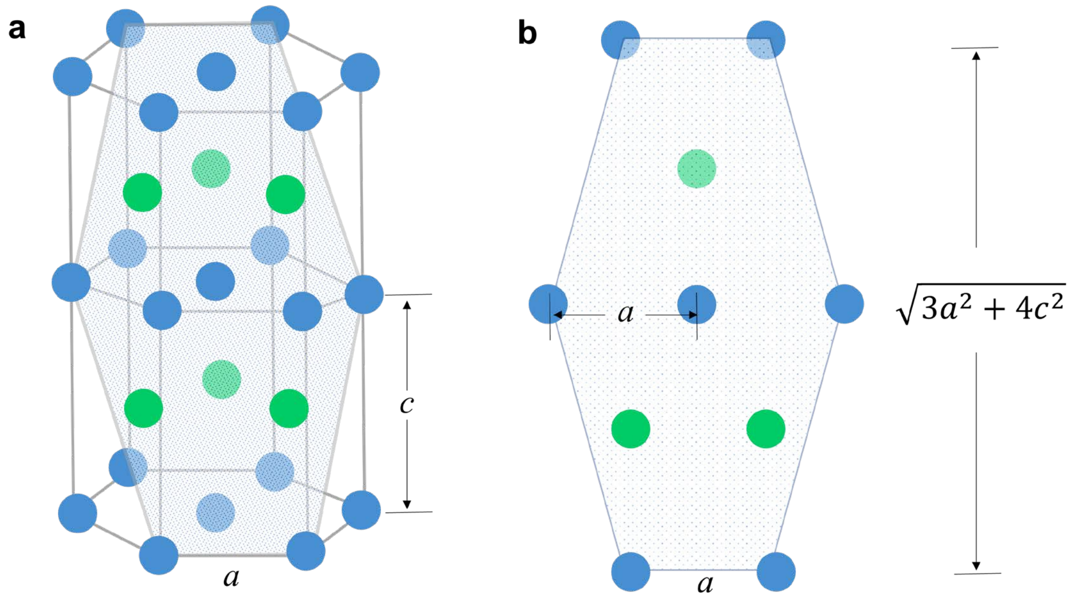

Supplementary Fig. 13 | Model of the *hcp* phase and the {01-11} plane. (a) A {01-11} plane in an *hcp* structure. Two unit-cells are included in this model. (b) Atoms exposed on the {01-11} plane. Blue and green atoms indicate *a* and *b* atoms in the *ababab* sequence, respectively.

In (b), the area was occupied by 3 blue atoms (6 at the corners × 1/3 + 1 at the center) and 3 green atoms, thus 6 in total.

The area of (b) can be calculated as:

$$A = 1/2 \times 3a \times \sqrt{3a^2 + 4c^2} = 43.29\ \text{Å}^2 = 4.329 \times 10^{-19}\ \text{m}^2 \qquad \text{(eq. 3)}$$

The area of occupied by each atom (A_0) can be calculated as:

$$A_0 = A/6 = 7.21 \times 10^{-20}\ \text{m}^2$$

Given 1 g of Pt, the number of Pt atoms on the surface of {01-11} facet can be calculated as (ignoring the *fcc*-Pt at the top of the nanobranches):

$$N_{\text{surface}} = 1\ \text{g} / (195.1\ \text{g mol}^{-1}) \times 6.02 \times 10^{23}\ \text{mol}^{-1} / n = 3.09 \times 10^{21} / n$$

n: atomic layer number of Pt

The surface area can be calculated as:

$$\text{Surface area} = N_{\text{surface}}\, A_0 = 3.09 \times 10^{21} \times A_0 / n = 222.8/n\ (\text{m}^2)$$

The specific surface area is

$$\text{ECSA} = 222.8/n\ (\text{m}^2\ \text{g}^{-1}) \qquad \text{(eq. 4)}$$

This result, thus, quantifies the k value in eq. 1, i.e., $k = 222.8\ \text{m}^2\ \text{g}^{-1}$.

The ECSAs and specific activities of the *hcp*-Ni@Pt_{nL} nanobranches in the HER can be calculated by eq. 4, as summarized in Supplementary Table 2.

Supplementary Table 2 | ECSAs and specific activities of the *hcp*-Ni@Pt$_{n\mathrm{L}}$ nanobranches in the alkaline HER, compared with those of Pt/C.

Sample	ECSA (m^2 g^{-1})	Specific activity at –70 mV (mA cm^{-2})
hcp-Ni@Pt$_{1.3L}$	171	1.31
hcp-Ni@Pt$_{2.6L}$	85.7	20.3
hcp-Ni@Pt$_{3.2L}$	69.6	17.3
hcp-Ni@Pt$_{4.0L}$	55.7	14.3
Pt/C	54.6	2.0

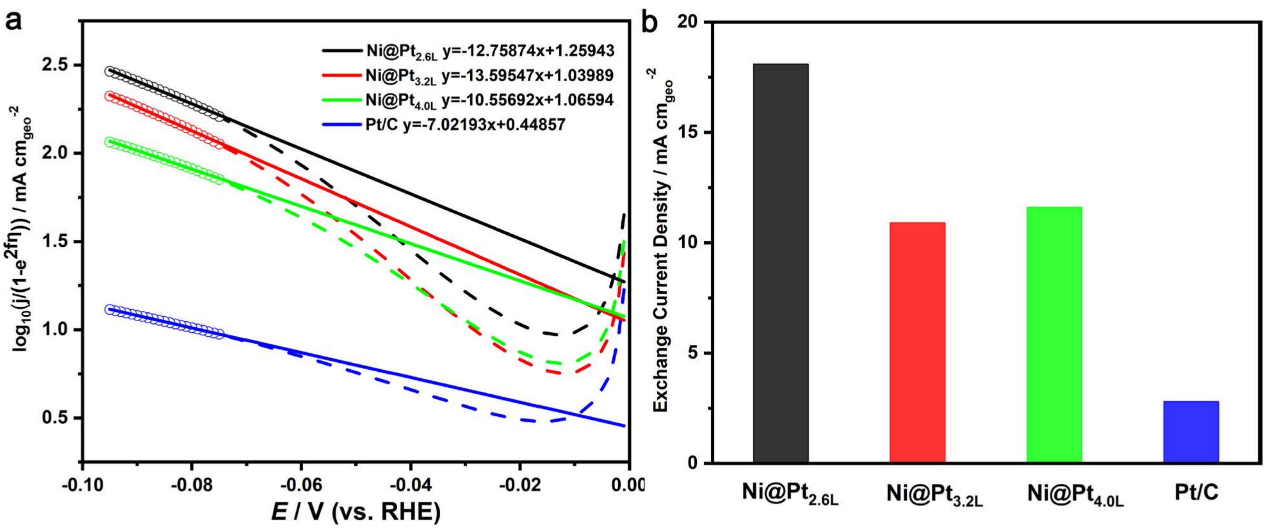

Supplementary Fig. 14 | Calculation of the exchange current densities with different catalysts. The current densities were normalized to the geometric area of the electrodes. The plots were derived from the LSV curves in Figure 4b (Experimental conditions: 1.5 µg Pt, 1 M KOH, 90% *iR* compensation). We consider an HER process containing two electron-transfer steps (number of transferred electrons, n = 2). The Butler-Volmer equation is described as $j = j_0[e^{-(n'+\alpha)f\eta} - e^{(n''+1-\alpha)f\eta}]$, where j is the current density, j_0 is the exchange current density, α is the transfer coefficient, $f = F/RT$ is a constant, η is the overpotential, n' and n'' are the numbers of the transferred electrons before and after the rate-determining step, $n'+n''+1 = n$. This equation can be changed to $j = j_0\, e^{-(n'+\alpha)f\eta}\,(1-e^{nf\eta})$ and further to $\log_{10}[j/(1-e^{nf\eta})] = \log_{10} j_0 - [(n'+\alpha)F/(2.303RT)]\,\eta$. Therefore, the exchange current density (j_0) can be derived by plotting $\log_{10}[j/(1-e^{nf\eta})]$ against η. (a) The $\log_{10}[j/(1-e^{nf\eta})] \sim \eta$ plots with different catalysts. (b) The calculated exchange current densities based on the polarization curves in the potential range of –75 ~ –95 mV. The exchange current density of the $Ni@Pt_{2.6L}$ was calculated to be 18.2 mA cm_{geo}^{-2}, which is 6.5 times greater than that of the commercial Pt/C (2.8 mA cm_{geo}^{-2}), confirming the boosted kinetics of the HER with this catalyst.

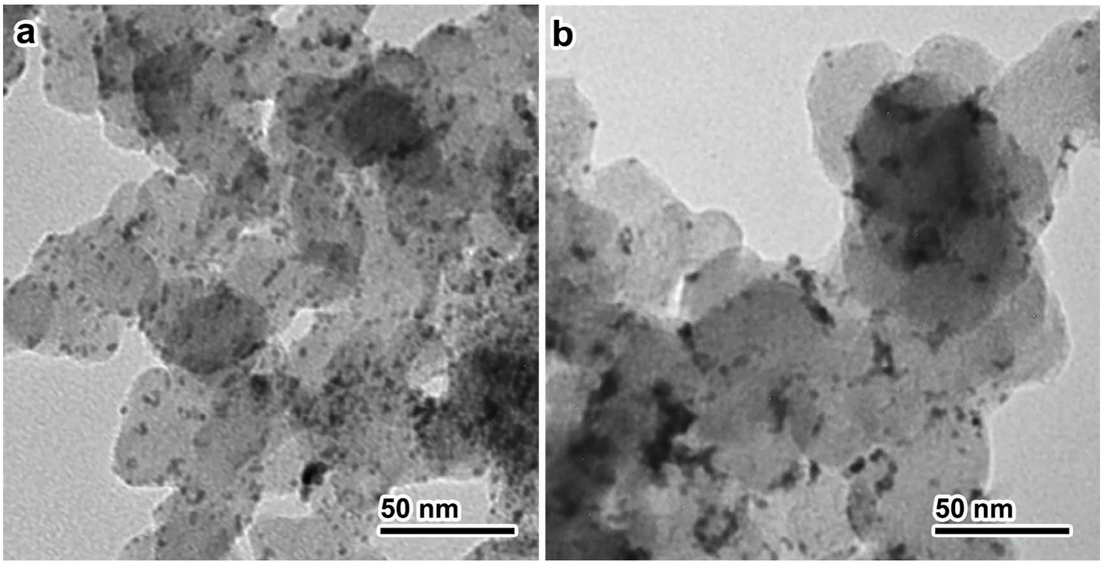

Supplementary Fig. 15 | Characterization of the commercial Pt/C before and after the stability test. The stability test was carried out by chronopotentiometry at 10 mA cm_{geo}^{-2} for 10 h. (a, b) TEM images of the Pt/C before and after the stability test, respectively. Significant aggregation of the Pt nanoparticles can be observed during the catalysis.

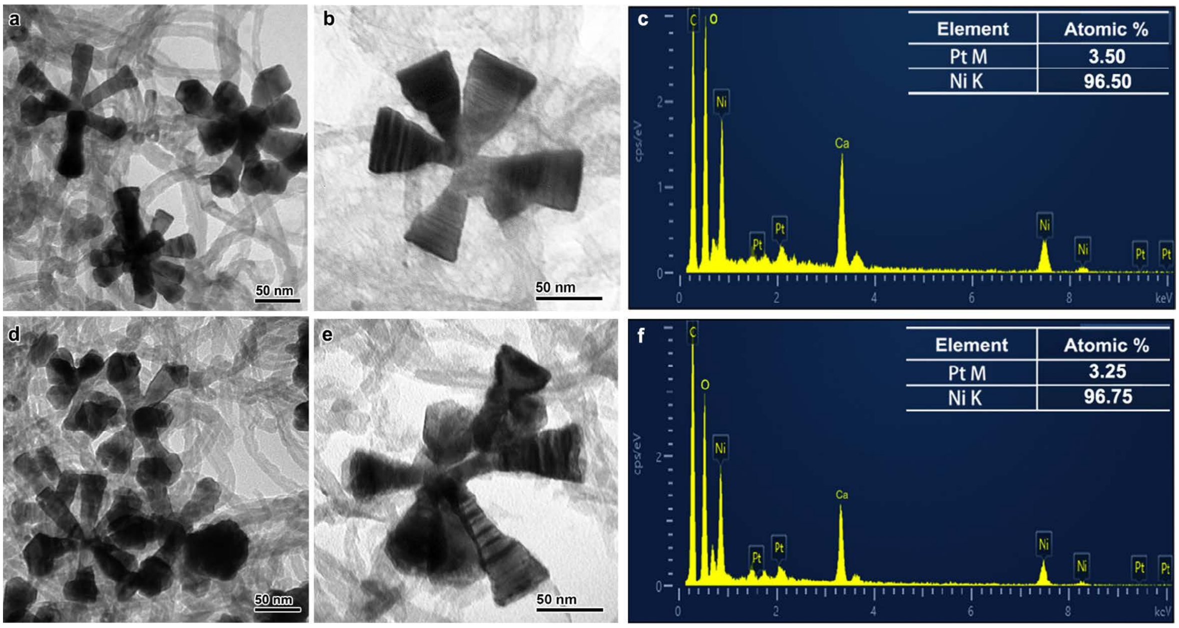

Supplementary Fig. 16 | Stability of the *hcp*-Ni@Pt$_{2.6L}$ nanobranches during the chronopotentiometric stability test at 10 mA cm$_{geo}^{-2}$ for 60 h. (a, b) TEM images of the nanobranches before the stability test. (c) EDS of the nanobranches before the stability test. (d, e) TEM images of the nanobranches after the stability test. (f) EDS of the nanobranches after the stability test. No obvious morphological/compositional changes can be detected, confirming the integrity of the core-shell nanostructure during the HER.

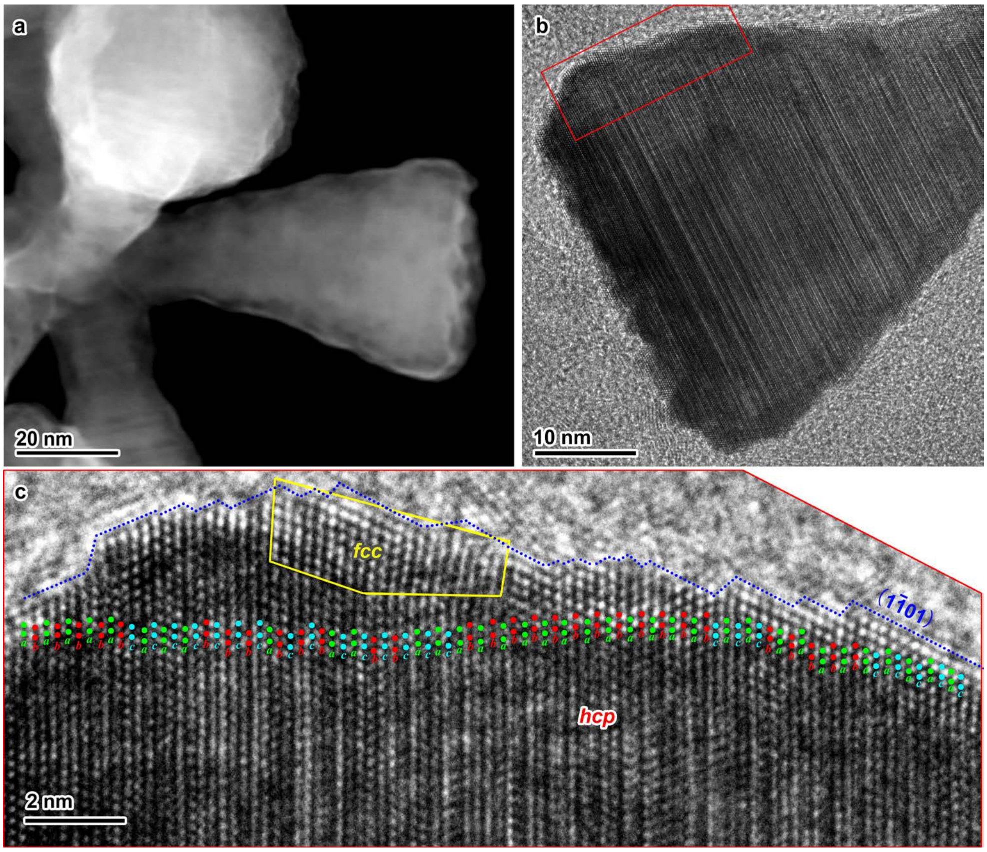

Supplementary Fig. 17 | Structure analysis of the *hcp*-Ni@Pt$_{2.6L}$ core-shell nanobranches after the chronopotentiometric stability test (current density, 10 mA cm^{-2}; duration, 6 h). (a, b) HAADF-STEM and HRTEM images, respectively. (c) An enlarged HRTEM image of the rectangle-indicated area of (b). Parts of the metastable *hcp*-Pt skin transformed into the stable *fcc* phase after the stability test.

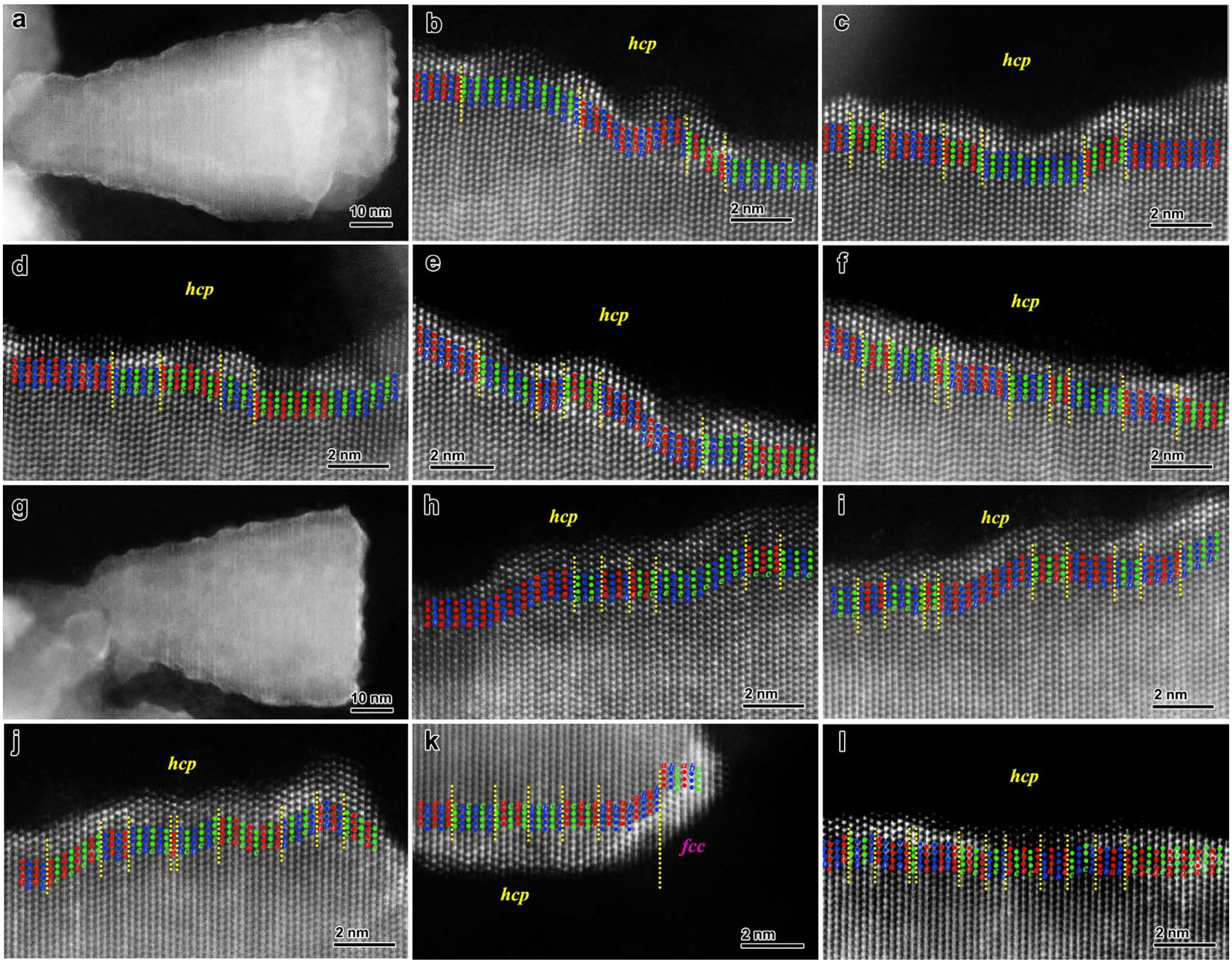

Supplementary Fig. 18 | Structural analysis of the *hcp*-Ni@Pt$_{2.6L}$ core-shell nanobranches after the chronopotentiometric stability test at 100 mA cm^{-2} for 20 h. Two nanobranches were examined domain by domain. (a, g) HAADF-STEM images of the respective nanobranches. (b–f, h–l) C_s-corrected high-resolution HAADF-STEM images. The Pt-skins on the sides of the nanobranches have retained the metastable *hcp* phase. The {1-101} facets and step sites were also retained. At the tip of the nanobranch, Pt-skin retained its *fcc* phase (k). It can be concluded that the *hcp*-Ni@Pt nanobranches showed high structural stability during the electrocatalytic HER. It may be because the phase change involves the position change of a large number of atoms, which is a difficult process with a high energy barrier. Therefore, the phase change can only be observed occasionally in small local areas as shown in Supplementary Fig. 17. By statistically analyzing all the high-resolution images available, including the 10 images in Supplementary Fig. 18 and the one image in Supplementary Fig. 17, the faction of the phase change during the catalysis can be roughly estimated to be ~3% (*hcp*, 635 atomic lines; *fcc*, 21 atomic lines).

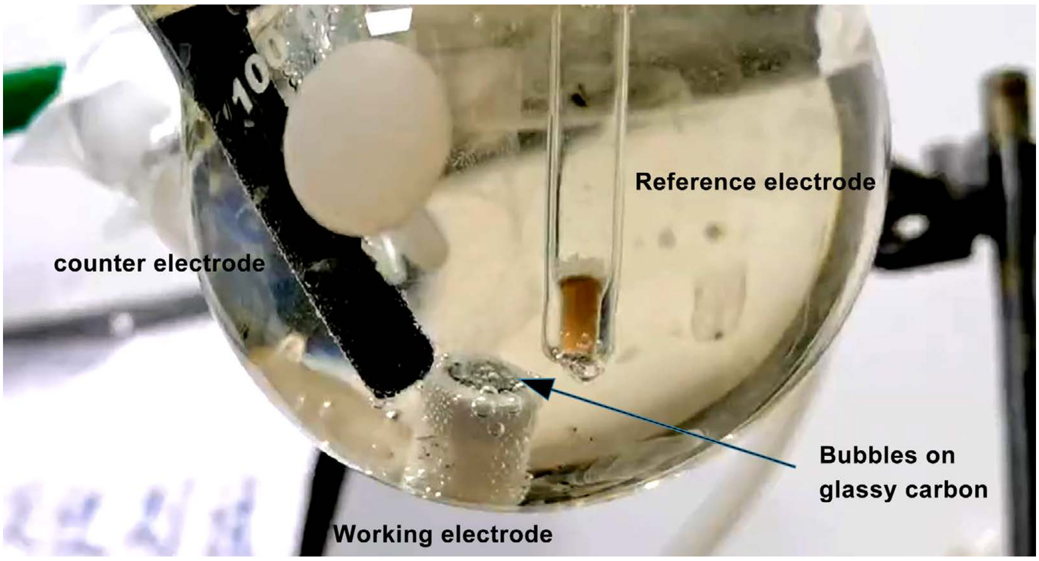

Supplementary Fig. 19 | A photo of the three-electrode setup during the chronopotentiometric stability test at 100 mA cm^{-2}. The H_2 bubbles on the working electrode can be clearly observed, which may partially block the active sites of the catalyst.

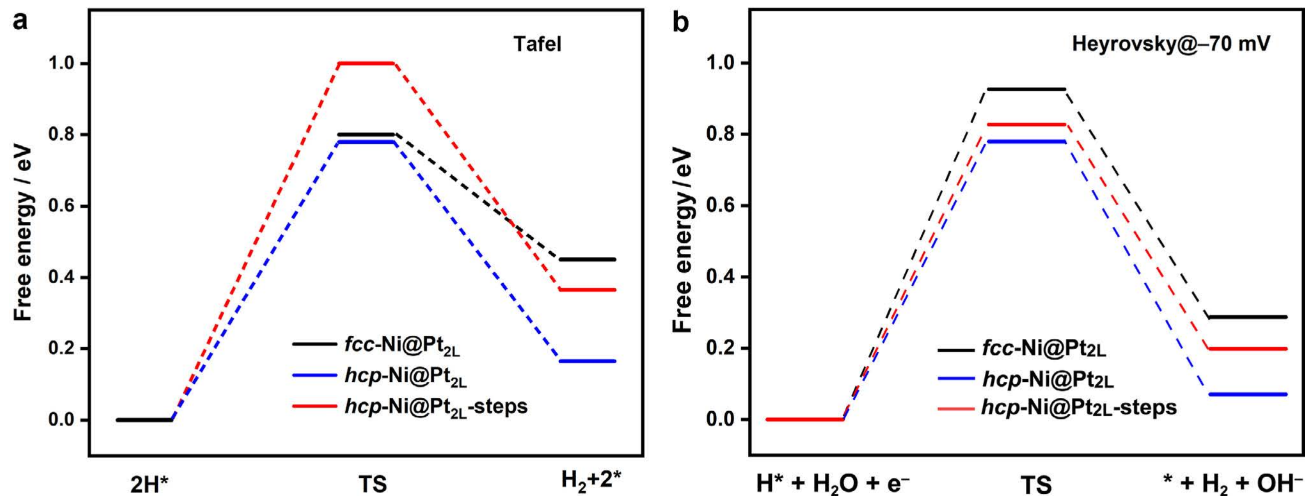

Supplementary Fig. 20 | Free energy diagrams of the Tafel reaction (a) and the Heyrovsky reaction (b) at –70 mV on *hcp*-Ni@Pt$_{2L}$, *hcp*-Ni@Pt$_{2L}$ with step sites, and *fcc*-Ni@Pt$_{2L}$ surfaces. On the *fcc*-Ni@Pt$_{2L}$ surface, the energy barriers for the Tafel reaction and the Heyrovsky reaction were almost identical. Considering that the Heyrovsky reaction involves the solvent of H_2O in large excess as a reactant, it may become a more favorable process for hydrogen desorption, which is consistent with the experimental Tafel slope of 51 mV dec^{-1}. The results in (b) also indicate that the metastable *hcp* phase contributes to the decreased energy barrier of the Heyrovsky reaction, compared with the *fcc* counterpart.

Supplementary Table 3 | Comparison of the reaction barriers in our results with those reported in the literature.

Systems	Energy Barrier / eV	Reaction Types	Reference
hcp-Ni@Pt_{2L}-steps	0.63/0.83	Volmer/Heyrovsky	This Work
PtNi alloy	0.50–0.80	Volmer	6
Ru(111)	0.68/0.96	Volmer/Heyrovsky	16
Rh(111)	0.95/1.12	Volmer/Heyrovsky	16
Pt(111)	0.80/1.02	Volmer/Heyrovsky	16
RhRu alloy	0.45/0.75	Volmer/Heyrovsky	16
TMs-Ti_2C	0.5–1.5	Heyrovsky	17

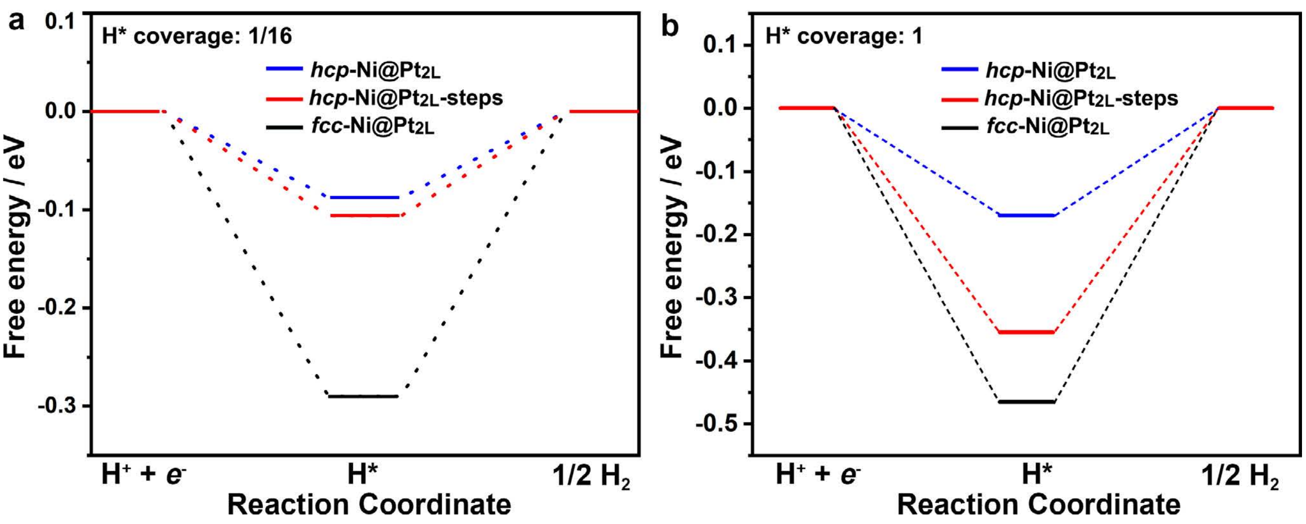

Supplementary Fig. 21 | Binding energies of hydrogen on *hcp*-Ni@Pt$_{2L}$, *hcp*-Ni@Pt$_{2L}$ with step sites, and *fcc*-Ni@Pt$_{2L}$ surfaces at low (a, H* coverage = 1/16) and high (b, H* coverage = 1) coverages of H*. The DFT results suggest that the coverage of H* on the catalyst surface can affect the binding energy of hydrogen. However, the general trend is similar. In both calculations, the binding energy of hydrogen on *hcp*-Ni@Pt$_{2L}$ was closest to 0, indicating the best HER performance, consistent with experimental observations.

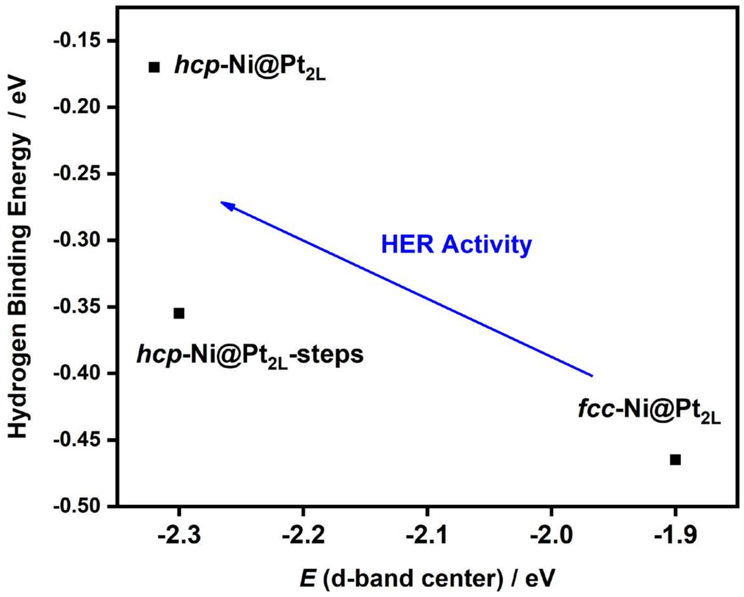

Supplementary Fig. 22 | DFT-calculated relationship between the *d*-band center and the hydrogen binding energy (ΔG_{H*}). The *d*-band centers of *fcc*-Ni@Pt_{2L}, *hcp*-Ni@Pt_{2L}-steps, and *hcp*-Ni@Pt_{2L} are –1.89, –2.30, and –2.32 eV, respectively. It can be inferred that the trend of the *d*-band center values is roughly correlated with the trend of the hydrogen binding energy values. According to the *d*-band center theory, the metastable *hcp* catalysts weakly adsorb guest molecules, corresponding to low hydrogen binding energies, which promises improved HER performance.

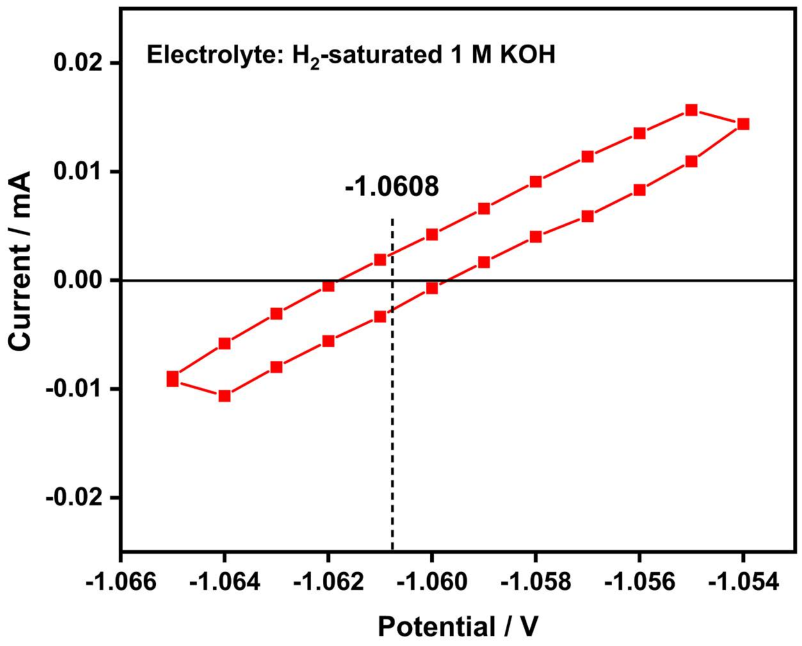

Supplementary Fig. 23 | Calibration of the SCE reference electrode. We adopted a three-electrode system by using a freshly polished Pt wire, a graphite electrode, and a saturated calomel electrode (SCE, to be calibrated) as the working, counter, and reference electrode, respectively. The electrolyte was 1 M KOH saturated by H_2. The temperature was maintained at 25°C. CVs were run at a scan rate of 1 mV s^{-1}, and the average of the two potentials at which the current crossed zero was taken to be the thermodynamic potential for the hydrogen electrode reactions (i.e., 0 V by definition)[18]. The average potential value, thus, is the negative potential value of the reference electrode vs. RHE. This figure shows CV curves of a Pt wire in H_2-saturated 1 M KOH at 25 °C. The SCE electrode potential was determined to be 1.0608 V vs. RHE, which is very close to the calculated value by Nernst equation (1.0574 V, i.e., 0.2412 + 0.0591 pH, pH = 13.81, see Supplementary Fig. 24). The calibration was carried out before each electrocatalytic measurement.

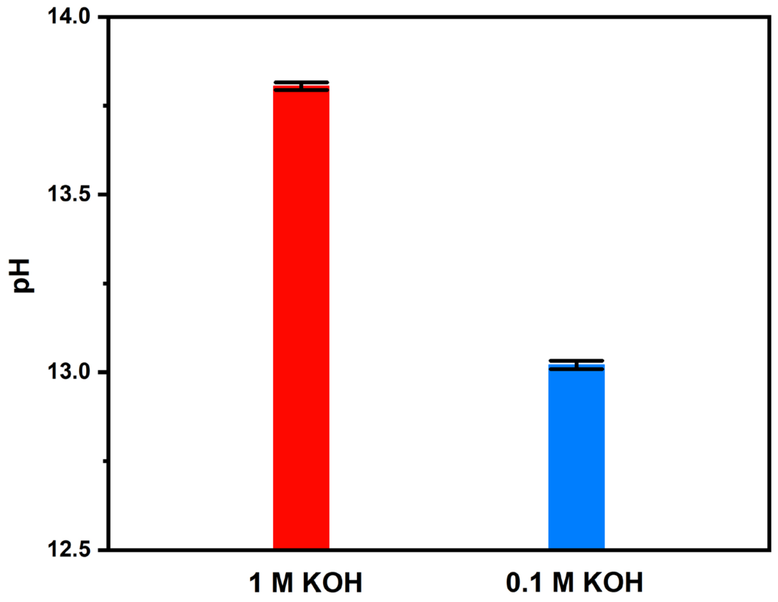

Supplementary Fig. 24 | pH of the electrolytes for the HER. The pH of 1 M KOH and 0.1 M KOH were measured to be 13.81 ± 0.01 and 13.02 ± 0.01, respectively, on a pH-meter (Sartorius PB-10). The error bars indicate the standard deviations of the pH values from 10 parallel measurements.

Supplementary Table 4 | Comparison of the hydrogen binding energies on a benchmark calculated with different DFT functionals.

Functional	Hydrogen Binding Energy / eV
PBE	–0.50
B3LYP	–0.53
PBE0	–0.76

Coordinates of atoms for Fig. 4c in VASP format:

H on *hcp*-Ni@Pt (1% strain)

```
  1.0
     7.5697999999999999     0.0000000000000000     0.0000000000000000
    -0.0000579600000000     8.3016120000000004     0.0000000000000000
    -0.0001695400000000    -0.0001695500000000    24.2853999999999992
  Ni   Pt   H
   30   24   1
Selective dynamics
Direct
  0.9998799999999974  0.3759700000000024  0.0164699999999982   F   F   F
  0.9998799999999974  0.3740681800000019  0.1918432200000026   T   T   T
  0.1665500000000009  0.3759700000000024  0.1047000000000011   F   F   F
  0.9998799999999974  0.1259700000000024  0.1341099999999997   F   F   F
  0.1665500000000009  0.1259700000000024  0.0458799999999968   F   F   F
  0.1665500000000009  0.1429968200000005  0.2240905800000021   T   T   T
  0.3332100000000011  0.3759700000000024  0.0164699999999982   F   F   F
  0.3332100000000011  0.3740681800000019  0.1918432200000026   T   T   T
  0.4998799999999974  0.3759700000000024  0.1047000000000011   F   F   F
  0.3332100000000011  0.1259700000000024  0.1341099999999997   F   F   F
  0.4998799999999974  0.1259700000000024  0.0458799999999968   F   F   F
  0.4998799999999974  0.1429968200000005  0.2240905800000021   T   T   T
  0.6665500000000009  0.3759700000000024  0.0164699999999982   F   F   F
  0.6665500000000009  0.3740681800000019  0.1918432200000026   T   T   T
  0.8332100000000011  0.3759700000000024  0.1047000000000011   F   F   F
  0.6665500000000009  0.1259700000000024  0.1341099999999997   F   F   F
  0.8332100000000011  0.1259700000000024  0.0458799999999968   F   F   F
  0.8332100000000011  0.1429968200000005  0.2240905800000021   T   T   T
  0.9998799999999974  0.8759700000000024  0.0164699999999982   F   F   F
  0.1665500000000009  0.8759700000000024  0.1047000000000011   F   F   F
  0.9998799999999974  0.6259700000000024  0.1341099999999997   F   F   F
  0.1665500000000009  0.6259700000000024  0.0458799999999968   F   F   F
  0.3332100000000011  0.8759700000000024  0.0164699999999982   F   F   F
  0.4998799999999974  0.8759700000000024  0.1047000000000011   F   F   F
  0.3332100000000011  0.6259700000000024  0.1341099999999997   F   F   F
  0.4998799999999974  0.6259700000000024  0.0458799999999968   F   F   F
  0.6665500000000009  0.8759700000000024  0.0164699999999982   F   F   F
  0.8332100000000011  0.8759700000000024  0.1047000000000011   F   F   F
  0.6665500000000009  0.6259700000000024  0.1341099999999997   F   F   F
  0.8332100000000011  0.6259700000000024  0.0458799999999968   F   F   F
  0.9999158059611988  0.2994416168059890  0.4196187314501229   T   T   T
  0.1665500000000009  0.4799716099999998  0.3390489100000025   T   T   T
  0.9998799999999974  0.1928410700000001  0.3134560800000017   T   T   T
```

0.1665354351296621	0.0142330009039361	0.4066159290100988	T	T	T
0.3331076119599740	0.3003468611912748	0.4196741732126403	T	T	T
0.4998799999999974	0.4799716099999998	0.3390489100000025	T	T	T
0.3332100000000011	0.1928410700000001	0.3134560800000017	T	T	T
0.4998630490572227	0.0163873077427507	0.4053517366958093	T	T	T
0.6667238199156439	0.3002380863828925	0.4196776984935198	T	T	T
0.8332100000000011	0.4799716099999998	0.3390489100000025	T	T	T
0.6665500000000009	0.1928410700000001	0.3134560800000017	T	T	T
0.8331740795379825	0.0141637029175824	0.4067251721331495	T	T	T
0.9998799999999974	0.8782723500000031	0.1969701000000015	T	T	T
0.1673342021808156	0.8900670069699926	0.2976042476090673	T	T	T
-0.0000716983436418	0.7368259332807898	0.3819868712072519	T	T	T
0.1665500000000009	0.6023098800000000	0.2386974400000028	T	T	T
0.3332100000000011	0.8782723500000031	0.1969701000000015	T	T	T
0.4999318894973032	0.8912034145938200	0.2969104876219598	T	T	T
0.3309880688356113	0.7329197945725521	0.3867515083281959	T	T	T
0.4998799999999974	0.6023098800000000	0.2386974400000028	T	T	T
0.6665500000000009	0.8782723500000031	0.1969701000000015	T	T	T
0.8325418342285171	0.8902026177169524	0.2976187967834872	T	T	T
0.6688275672582089	0.7329728857458103	0.3867054518337908	T	T	T
0.8332100000000011	0.6023098800000000	0.2386974400000028	T	T	T
0.5001356712278143	0.6865639974552101	0.4329116162689015	T	T	T

H on *hcp*-Ni@Pt (3% strain)

1.0

7.4966609999999996	0.0000000000000000	0.0000000000000000
-0.0000574000000000	8.2214010000000002	0.0000000000000000
-0.0001695400000000	-0.0001695500000000	24.2853999999999992

Ni Pt H

30 24 1

Selective dynamics

Direct

0.9998799999999974	0.3759700000000024	0.0164699999999982	F	F	F
0.9998799999999974	0.3740681800000019	0.1918432200000026	T	T	T
0.1665500000000009	0.3759700000000024	0.1047000000000011	F	F	F
0.9998799999999974	0.1259700000000024	0.1341099999999997	F	F	F
0.1665500000000009	0.1259700000000024	0.0458799999999968	F	F	F
0.1665500000000009	0.1429968200000005	0.2240905800000021	T	T	T
0.3332100000000011	0.3759700000000024	0.0164699999999982	F	F	F
0.3332100000000011	0.3740681800000019	0.1918432200000026	T	T	T
0.4998799999999974	0.3759700000000024	0.1047000000000011	F	F	F
0.3332100000000011	0.1259700000000024	0.1341099999999997	F	F	F

0.4998799999999974	0.1259700000000024	0.0458799999999968	F	F	F
0.4998799999999974	0.1429968200000005	0.2240905800000021	T	T	T
0.6665500000000009	0.3759700000000024	0.0164699999999982	F	F	F
0.6665500000000009	0.3740681800000019	0.1918432200000026	T	T	T
0.8332100000000011	0.3759700000000024	0.1047000000000011	F	F	F
0.6665500000000009	0.1259700000000024	0.1341099999999997	F	F	F
0.8332100000000011	0.1259700000000024	0.0458799999999968	F	F	F
0.8332100000000011	0.1429968200000005	0.2240905800000021	T	T	T
0.9998799999999974	0.8759700000000024	0.0164699999999982	F	F	F
0.1665500000000009	0.8759700000000024	0.1047000000000011	F	F	F
0.9998799999999974	0.6259700000000024	0.1341099999999997	F	F	F
0.1665500000000009	0.6259700000000024	0.0458799999999968	F	F	F
0.3332100000000011	0.8759700000000024	0.0164699999999982	F	F	F
0.4998799999999974	0.8759700000000024	0.1047000000000011	F	F	F
0.3332100000000011	0.6259700000000024	0.1341099999999997	F	F	F
0.4998799999999974	0.6259700000000024	0.0458799999999968	F	F	F
0.6665500000000009	0.8759700000000024	0.0164699999999982	F	F	F
0.8332100000000011	0.8759700000000024	0.1047000000000011	F	F	F
0.6665500000000009	0.6259700000000024	0.1341099999999997	F	F	F
0.8332100000000011	0.6259700000000024	0.0458799999999968	F	F	F
-0.0000355169678088	0.3035153524187826	0.4211913467283938	T	T	T
0.1665500000000009	0.4799716099999998	0.3390489100000025	T	T	T
0.9998799999999974	0.1928410700000001	0.3134560800000017	T	T	T
0.1667785459904364	0.0158999551182706	0.4113499116154402	T	T	T
0.3331004153876743	0.3066935816876296	0.4212228766710230	T	T	T
0.4998799999999974	0.4799716099999998	0.3390489100000025	T	T	T
0.3332100000000011	0.1928410700000001	0.3134560800000017	T	T	T
0.4999185694475911	0.0211854335320113	0.4026801737220804	T	T	T
0.6668425926478582	0.3066489217777509	0.4212006026908262	T	T	T
0.8332100000000011	0.4799716099999998	0.3390489100000025	F	F	F
0.6665500000000009	0.1928410700000001	0.3134560800000017	F	F	F
0.8330594064339920	0.0159237312579404	0.4113613622726037	T	T	T
0.9998799999999974	0.8782723500000031	0.1969701000000015	T	T	T
0.1672280442266763	0.8883303822134575	0.2993327131556949	T	T	T
-0.0000717283985434	0.7374863197266076	0.3847094080485154	T	T	T
0.1665500000000009	0.6023098800000000	0.2386974400000028	T	T	T
0.3332100000000011	0.8782723500000031	0.1969701000000015	T	T	T
0.4999293092038918	0.8885144868369141	0.2968845221874194	T	T	T
0.3310619222976960	0.7320046052977940	0.3895198483386936	T	T	T
0.4998799999999974	0.6023098800000000	0.2386974400000028	T	T	T
0.6665500000000009	0.8782723500000031	0.1969701000000015	T	T	T
0.8327213040332928	0.8883076815321945	0.2993286457517619	T	T	T
0.6688185441319995	0.7321176652803836	0.3894520516664025	T	T	T
0.8332100000000011	0.6023098800000000	0.2386974400000028	T	T	T

0.4997739760136004 0.6866938678743947 0.4364326545436579 T T T

H on *hcp*-Ni@Pt (5% strain)

1.0
7.4227999999999996 0.0000000000000000 0.0000000000000000
-0.0000284200000000 8.1403999999999996 0.0000000000000000
-0.0000847700000000 -0.0000847700000000 24.2853999999999992
Ni Pt H
30 24 1
Selective dynamics
Direct
0.9998799999999974 0.3759700000000024 0.0164699999999982 F F F
0.9997422426516867 0.3757437298988059 0.1920834213641635 T T T
0.1665500000000009 0.3759700000000024 0.1047000000000011 F F F
0.9998799999999974 0.1259700000000024 0.1341099999999997 F F F
0.1665500000000009 0.1259700000000024 0.0458799999999968 F F F
0.1638616758398873 0.1422815376437841 0.2238724402575044 T T T
0.3332100000000011 0.3759700000000024 0.0164699999999982 F F F
0.3305480786579691 0.3738202638930420 0.1918723438632048 T T T
0.4998799999999974 0.3759700000000024 0.1047000000000011 F F F
0.3332100000000011 0.1259700000000024 0.1341099999999997 F F F
0.4998799999999974 0.1259700000000024 0.0458799999999968 F F F
0.4996419917053925 0.1434309936258805 0.2222832929362401 T T T
0.6665500000000009 0.3759700000000024 0.0164699999999982 F F F
0.6688852528930863 0.3738708459693390 0.1918704577418066 T T T
0.8332100000000011 0.3759700000000024 0.1047000000000011 F F F
0.6665500000000009 0.1259700000000024 0.1341099999999997 F F F
0.8332100000000011 0.1259700000000024 0.0458799999999968 F F F
0.8353470575794416 0.1423366493815104 0.2238692853985971 T T T
0.9998799999999974 0.8759700000000024 0.0164699999999982 F F F
0.1665500000000009 0.8759700000000024 0.1047000000000011 F F F
0.9998799999999974 0.6259700000000024 0.1341099999999997 F F F
0.1665500000000009 0.6259700000000024 0.0458799999999968 F F F
0.3332100000000011 0.8759700000000024 0.0164699999999982 F F F
0.4998799999999974 0.8759700000000024 0.1047000000000011 F F F
0.3332100000000011 0.6259700000000024 0.1341099999999997 F F F
0.4998799999999974 0.6259700000000024 0.0458799999999968 F F F
0.6665500000000009 0.8759700000000024 0.0164699999999982 F F F
0.8332100000000011 0.8759700000000024 0.1047000000000011 F F F
0.6665500000000009 0.6259700000000024 0.1341099999999997 F F F
0.8332100000000011 0.6259700000000024 0.0458799999999968 F F F
-0.0008462037690212 0.3041872538588716 0.4236347954208312 T T T
0.1656700223915032 0.4802352469042427 0.3400114151385711 T T T

-0.0005985933790768	0.1899601018338577	0.3152456523414264	T	T	T
0.1673179990299833	0.0147597233195653	0.4183563773208180	T	T	T
0.3330812660845476	0.3094429816606658	0.4206040461328114	T	T	T
0.4992382570593493	0.4839287563643599	0.3363779671077083	T	T	T
0.3322347303281807	0.1984394468518916	0.3106653130297235	T	T	T
0.4990826529953520	0.0214245318180750	0.3986722889756456	T	T	T
0.6651419334295049	0.3094164272347835	0.4205765765551346	T	T	T
0.8328922832633960	0.4801959454923086	0.3400028697145592	T	T	T
0.6665766561478383	0.1983276676556698	0.3107086846414941	T	T	T
0.8309399609886077	0.0148980287780974	0.4181818975813080	T	T	T
-0.0002904621279021	0.8772524699095953	0.1975583957411505	T	T	T
0.1668863206049503	0.8883514688968805	0.3026061284442949	T	T	T
-0.0009158859434957	0.7373631043946214	0.3882337161459753	T	T	T
0.1664005700179225	0.6041234797470602	0.2402438048361760	T	T	T
0.3319942362798934	0.8783161062564719	0.1969381129199960	T	T	T
0.4993203248969997	0.8862832082830789	0.2958705745251791	T	T	T
0.3304539606428198	0.7287091758633463	0.3935371120508324	T	T	T
0.4996758625856310	0.6027785176092180	0.2355165017823858	T	T	T
0.6674068416147148	0.8782982505862836	0.1969743070185504	T	T	T
0.8318064122284999	0.8883659833769039	0.3025995540376580	T	T	T
0.6676923599101123	0.7288386872549349	0.3933889177130535	T	T	T
0.8329524752793676	0.6041248773143135	0.2402369750587228	T	T	T
0.4993312696090141	0.6793764046818797	0.4406346771817278	T	T	T

References

1. Zhang, Q. *et al.* Crystal structure control of binary and ternary solid-solution alloy nanoparticles with a face-centered cubic or hexagonal close-packed phase. *J. Am. Chem. Soc.* **144**, 4224-4232 (2022).
2. Chen, Q. *et al.* Synergistic effect in ultrafine PtNiP nanowires for highly efficient electrochemical hydrogen evolution in alkaline electrolyte. *Appl. Catal., B* **301**, 120754 (2022).
3. Ding, J., Ji, Y., Li, Y. & Hong, G. Monoatomic platinum-embedded hexagonal close-packed nickel anisotropic superstructures as highly efficient hydrogen evolution catalyst. *Nano Lett.* **21**, 9381-9387 (2021).
4. Shi, Y. *et al.* Electronic metal-support interaction modulates single-atom platinum catalysis for hydrogen evolution reaction. *Nat. Commun.* **12**, 3021 (2021).
5. Nairan, A. *et al.* Proton selective adsorption on Pt-Ni nano-thorn array electrodes for superior hydrogen evolution activity. *Energy Environ. Sci.* **14**, 1594-1601 (2021).
6. Zhang, C. *et al.* H_2 in situ inducing strategy on Pt surface segregation over low Pt doped $PtNi_5$ nanoalloy with superhigh alkaline HER activity. *Adv. Funct. Mater.* **31**, 202008298 (2021).
7. Alinezhad, A. *et al.* Controlling hydrogen evolution reaction activity on Ni core-Pt island nanoparticles by tuning the size of the Pt islands. *Chem. Commun.* **57**, 2788-2791 (2021).
8. Yang, M., Zhang, J., Zhang, W., Wu, Z. & Gao, F. Pt nanoparticles/Fe-doped α-$Ni(OH)_2$ nanosheets array with low Pt loading as a high-performance electrocatalyst for alkaline hydrogen evolution reaction. *J. Alloys Compd.* **823**, 153790 (2020).
9. Li, M. *et al.* Single-atom tailoring of platinum nanocatalysts for high-performance multifunctional electrocatalysis. *Nat. Catal.* **2**, 495-503 (2019).

10. Alinezhad, A. *et al.* Direct growth of highly strained Pt islands on branched Ni nanoparticles for improved hydrogen evolution reaction activity. *J. Am. Chem. Soc.* **141**, 16202-16207 (2019).

11. Zhao, Z. *et al.* Surface-engineered PtNi-O nanostructure with record-high performance for electrocatalytic hydrogen evolution reaction. *J. Am. Chem. Soc.* **140**, 9046-9050 (2018).

12. Zhang, Z. *et al.* Crystal phase and architecture engineering of lotus-thalamus-shaped Pt-Ni anisotropic superstructures for highly efficient electrochemical hydrogen evolution. *Adv. Mater.* **30**, e1801741 (2018).

13. Liu, Z. *et al.* Aqueous synthesis of ultrathin platinum/non-noble metal alloy nanowires for enhanced hydrogen evolution activity. *Angew. Chem. Int. Ed.* **57**, 11678-11682 (2018).

14. Cao, Z. *et al.* Platinum-nickel alloy excavated nano-multipods with hexagonal close-packed structure and superior activity towards hydrogen evolution reaction. *Nat. Commun.* **8**, 15131 (2017).

15. Yin, H. *et al.* Ultrathin platinum nanowires grown on single-layered nickel hydroxide with high hydrogen evolution activity. *Nat. Commun.* **6**, 6430 (2015).

16. Li, Y. *et al.* Mesoporous RhRu nanosponges with enhanced water dissociation toward efficient alkaline hydrogen evolution. *ACS Appl. Mater. Interfaces* **13**, 5052-5060 (2021).

17. Gu, Y. *et al.* Single atom-modified hybrid transition metal carbides as efficient hydrogen evolution reaction catalysts. *Adv. Funct. Mater.* **31**, 2104285 (2021).

18. Liang, Y. *et al.* Co_3O_4 nanocrystals on graphene as a synergistic catalyst for oxygen reduction reaction. *Nature Materials* **10**, 780-786 (2011).
